# Hyperoxia-Induced Proliferative Retinopathy: Early Interruption of Retinal Vascular Development with Severe and Irreversible Neurovascular Disruption

**DOI:** 10.1371/journal.pone.0166886

**Published:** 2016-11-18

**Authors:** Michelle Lajko, Herminio J. Cardona, Joann M. Taylor, Ronil S. Shah, Kathryn N. Farrow, Amani A. Fawzi

**Affiliations:** 1 Department of Ophthalmology, Feinberg School of Medicine at Northwestern University, Chicago, Illinois, United States; 2 Department of Pediatrics, Feinberg School of Medicine at Northwestern University, Chicago, Illinois, United States; University of Utah (Salt Lake City), UNITED STATES

## Abstract

Bronchopulmonary dysplasia (BPD) is a major cause of neonatal morbidity in premature infants, occurring as a result of arrested lung development combined with multiple postnatal insults. Infants with BPD exposed to supplemental oxygen are at risk of retinopathy of prematurity as well. Thus, we studied the effects of hyperoxia on the retinal vasculature in a murine model of BPD. The retinal phenotype of this model, which we termed hyperoxia-induced proliferative retinopathy (HIPR), shows severe disruption of retinal vasculature and loss of vascular patterning, disorganized intra-retinal angiogenesis, inflammation and retinal detachment. Neonatal mice were subjected to 75% oxygen exposure from postnatal day (P)0 to P14 to model BPD, then allowed to recover in room air for 1 (P15), 7 (P21), or 14 days (P28). We quantified retinal thickness, protein levels of HIF-1α, NOX2, and VEGF, and examined the cellular locations of these proteins by immunohistochemistry. We examined the retinal blood vessel integrity and inflammatory markers, including macrophages (F4/80) and lymphocytes (CD45R). Compared to controls, normal retinal vascular development was severely disrupted and replaced by a disorganized sheet of intra-retinal angiogenesis in the HIPR mice. At all time-points, HIPR showed persistent hyaloidal vasculature and a significantly thinner central retina compared to controls. HIF-1α protein levels were increased at P15, while VEGF levels continued to increase until P21. Intra-retinal fibrinogen was observed at P21 followed by sub-retinal deposition in at P28. Inflammatory lymphocytes and macrophages were observed at P21 and P28, respectively. This model presents a severe phenotype of disrupted retinal vascular development, intra-retinal angiogenesis inflammation and retinal detachment.

## Introduction

Preterm birth, defined as birth at less than 37 weeks gestational age, accounts for about 10% of births in the US [1]. With advancements in neonatal care, the survival of extremely early gestational age (> 23 weeks) and low birth weight infants (< 1000g) has made bronchopulmonary dysplasia (BPD) the most common chronic lung disease and long-term morbidity affecting these preterm infants [2, 3]. With current day improved neonatal care and survival of smaller, more immature infants, the “new” BPD is quite different than in previous eras, and its hallmark is arrested lung and microvasculature development [3, 4].

Since normal retinal vascularization is incomplete until 36–40 weeks gestational age, premature infants are also susceptible to retinal vascular compromise [5]. Retinopathy of prematurity (ROP) is one of the leading causes of childhood blindness [6]. Originally, ROP was a direct consequence of excessive supplemental oxygen delivery, leading to impaired retinal vascular development, which, untreated, culminated in traction retinal detachment and visual impairment. With better control of the oxygen saturation in the neonatal units, the current day ROP is a confined to premature infants of gestational age less than 26 weeks old in the First World. In contrast, oxygen toxicity still remains a factor in the second world (third ROP epidemic) [7]. The incidence of ROP (stage 2 or worse) reaches 64% in preterm infants with a diagnosis of BPD and pulmonary hypertension [8]. Premature infants who receive supplemental oxygen in the setting of prematurity and lung underdevelopment may therefore be at risk for retinal vascular maldevelopment.

Rodent retinal vasculature develops entirely after birth, making them a good model to study vascular compromise associated with prematurity and oxygen exposure. One such model is the oxygen-induced retinopathy (OIR) where oxygen exposure is limited to 5 days, starting at day 7 postnatal (P7). OIR mice develop vaso-obliteration followed by pre-retinal neovascularization; however, the angiogenesis regresses and the retina revascularizes within 2–3 weeks [9, 10]. To model BPD, hyperoxia exposure to neonatal rodents replicates the anatomical (decreased alveolarization, increased collagen deposition, and increased interstitial thickness) and functional changes (decreased lung volume and elastance) seen in infants with BPD [11, 12]. While human infants with BPD are exposed to multiple other insults such as infection, inflammation, and poor nutrition that compound their lung disease, the hyperoxia-induced mouse model has been used extensively to investigate the specific role of oxidative injury in the development of BPD. Mice exposed to 85% oxygen after birth for 14 days, followed by room air recovery for 14 days have persistently abnormal lung development relative to age-matched controls raised in room air, suggesting that the oxidative insult is very slow to recover, if it recovers at all [13]. Rats, exposed to hyperoxia (75% oxygen) from P4 to P14, followed by 14 days in room air, have impaired lung aveolarization and a decrease in retinal thickness [14]. In the rat model of OIR, cycled between 50% and 10% oxygen for 14 days, preretinal neovascularization developed between the boundary of vascular and avascular retina [15].

In this study, we report the retinal phenotype in the hyperoxia-induced model of BPD in mice [16–18]. We explore the distinct and severe retinopathy that develops in this model, which we term hyperoxia-induced proliferative retinopathy (HIPR), focusing on angiogenesis, retinal thickness, blood vessel integrity, and inflammation. We focused our studies on the retinal vascular and oxidative changes in HIPR since the developing retinal vasculature is highly susceptible to oxygen perturbations [19]. Since hyperoxia exposure begins immediately after birth, we expected that HIPR would be significantly different from OIR. We hypothesized that normal vascular development will be severely affected and that the hyaloidal vasculature will persist to compensate for the deficient retinal vasculature. A major unknown was whether this severe hyperoxia-induced disruption of retinal vascular development would still be compatible with significant neuro-vascular recovery of these eyes in room air, similar to OIR, or whether these vascular changes would be so severe that they reach the retinal neurovascular developmental point of “no-return”.

## Methods

### Hyperoxia-Induced Proliferative Retinopathy Model

All animal procedures were performed in compliance with the recommendations in the *Guide for the Care and Use of Laboratory Animals of the National Institutes of Health*. All experimental protocols were approved by the Institutional Animal Care and Use Committee at Northwestern University. C57BL/6J (stock number: 000664, Jackson Laboratory, Bar Harbor, ME, USA) mice were placed in a Plexiglas chamber with an oxygen controller (Pro-Ox 110; Biospherix, Lacona, NY, USA) and exposed to 75% oxygen from birth to P14 [16, 17]. Exposure to hyperoxia was continuous, with brief interruptions only for animal care. Dams were rotated from hyperoxia to room air every 24 hours to prevent excessive oxygen toxicity [16]. Mice were euthanized after two weeks (P14) in high oxygen (HIPR) or moved to room air for 1 day (P15), 1 week (P21), or 2 weeks (P28) prior to euthanasia. Control, age-matched mice were raised in room air (RA). After experimental exposure, mouse pups were euthanized by isoflurane overdose and cervical dislocation. Subsequently, thoracotomy was performed to confirm death. Eyes were harvested prior to cervical dislocation. These procedures were carried out as recommended by the panel on euthanasia of the American Veterinary Medical Association. If study animals exhibited any signs of illness or distress, they would be immediately humanely euthanized. No animals utilized for this work became ill or died prior to the experimental endpoint.

### Retinal Flat Mounts

Whole eyes were fixed in 4% paraformaldehyde (Electron Microscopy Sciences, Hatfield, PA) diluted in phosphate buffered saline (PBS) for 3–4 hours. Eyes were stained as described previously [20]. Briefly, retinal cups were dissected and the vitreous removed. After several washes, the retinas were permeabilized with PBS/0.1% Triton X-100 for 18 hours. The retinas were blocked with 10% donkey serum, 1% Triton X-100, and 1% bovine serum albumin (BSA) for 2 hours. After washes, GS-Isolectin IB_4_ 594 (IB4) diluted in PBS with 1% Triton was applied for 18 hours (Table 1). After several washes, 4 radial incisions were made, and the retinas were mounted with ProLong Gold Antifade reagent (Thermo Fisher Scientific, Waltham, MA).

**Table 1:** List of antibodies used for immunohistochemistry and western blotting.

**Primary Antibodies**				
**Specificity**	**Source**	**Company**	**Dilution**	**Cell Type**
Glial fibrillary acidic protein (GFAP)	Rabbit	Abcam (ab7260)	1:200	Astrocytes [21], activated Mϋller cells [22]
HIF-1α	Rabbit	Cayman Chemicals (10006421)	1:500 WB	
β-actin	Mouse	Sigma-Aldrich (A5316)	1:5000	
HIF-1α	Rabbit	Abcam (ab2185)	1:100 IHC	
VEGF 164	Goat	R&D Biosystems (AF-493-NA)	1:80	EC, RGC, RPE, astrocytes, Mϋller cells [23]
CD31	Rat	BD Pharminogen (553370)	1:50	EC
VEGF receptor-2 (VEGFR2)	Rat	BD Pharminogen (555307)	1:100	EC [23, 24], Mϋller cells [25], PR [25], RGC [24], astrocytes [26]
gp91 [phox] (NOX2)	Mouse	BD Transduction Laboratories (611414)	1:200 IHC, 1:1000 WB	EC [27, 28]
Fibrinogen	Rabbit	Abcam (ab34269)	1:1000	Blood retinal barrier breakdown [29]
CD45R	Rat	AbD Serotec (MCA1258G)	1:300	Leukocytes [30, 31]
F4/80	Rat	AbD Serotec (MCA497G)	1:100	Macrophages [32]
Pax-6	Rabbit	BioLegend (901301)	1:200	Amacrine, horizontal, activated Mϋller cells [33]
**Secondary Antibodies**				
**Specificity**	**Conjugate**	**Source**	**Company**	**Dilution**
GS-isolectin IB_4_ (IB4)	Alexa Fluor 594		Thermo Fisher Scientific (I21413)	1:100
Rabbit	DyLight 488	Donkey	Abcam (ab96891)	1:200
Rabbit	HRP	Donkey	GE Healthcare (NA934)	1:5000
Mouse	HRP	Horse	Cell Signaling (7076)	1:5000
Rabbit	Biotin	Donkey	Jackson ImmunoResearch (711-065-152)	1:1000
Goat	Rhodamine Red	Donkey	Jackson ImmunoResearch (705-295-147)	1:200
Rat	Biotin	Goat	Jackson ImmunoResearch (112-065-167)	1:200
Streptavidin	Alexa Fluor 488		Thermo Fisher Scientific (S11223)	1:200
Rat	Alexa Fluor 594	Goat	Abcam (ab150160)	1:400
Mouse	Alexa Fluor 488	Donkey	Abcam (ab150105)	1:200
Rabbit	Alexa Fluor 647	Donkey	Abcam (ab150063)	1:300
NucBlue (DAPI)			Thermo Fisher Scientific (R37606)	-

Abbreviations: WB, western blotting. IHC, immunohistochemistry. EC, endothelial cells. PR, photoreceptors. RGC, retinal ganglion cells. RPE, retinal pigment epithelium.

### Immunohistochemistry

Retinal sections (7 μm) were deparaffinized and underwent antigen retrieval in sodium citrate buffer (10nM sodium citrate, 0.05% Tween-20, pH 6.0) at 80°C for 20 minutes. Sections were in blocking solution (10% donkey serum, 0.1% Triton X-100, 1% BSA) for 18 hours at 4°C. For HIF-1α staining, endogenous biotin was blocked with a streptavidin/biotin blocking kit (Vector Laboratories, Burlingame, CA), and endogenous peroxidase was blocked with 3% hydrogen peroxide for 15 minutes. Primary antibodies (Table 1) were applied for 1 hour in blocking solution. After washes, secondary antibodies were applied for 1 hour. For HIF-1α, ABC solution (Vector Laboratories) was applied for 30 minutes, visualized by 3,3’-diaminobenzidine (Sigma Aldrich), and counterstained with 0.5% methyl green (Sigma Aldrich). For all fluorescent antibodies (Table 1), sections were stained with 0.1% or 0.5% Sudan black in 70% ethanol to quench autofluorescence. Sections were counterstained with DAPI, rinsed, mounted, and sealed. Sections were imaged with a Zeiss LSM-510 Meta confocal microscope (Zeiss, Oberkochen, Germany) or with a Nikon 80i Eclipse upright microscope (Nikon, Tokyo, Japan) with a Photometrics CoolSnap CF camera (Photometrics, Tucson, AZ).

### Western Blotting

Retinas were processed as described previously [34]. Briefly, immediately after enucleation, retinas were dissected and lysed in 1X Mg2+ lysis buffer (EMD Millipore, Billerica, MA) with a protease inhibitor cocktail (Sigma Aldrich, St. Louis, MO) and a phosphatase inhibitor cocktail (EMD Millipore). Protein concentration of the supernatant was quantified using the Bradford method [35]. 40 μg of protein were separated on 4–20% Tris-glycine gel (Bio-Rad, Hercules, CA), transferred to nitrocellulose membrane, and blocked at room temperature with 5% BSA diluted in Tris-buffered saline supplemented with Tween-20 (TBST) for 1 hour. Membranes were incubated with primary antibodies (Table 1) diluted in 5% BSA in TBST for 18 hours at 4°C. After washing, membranes were incubated with secondary antibodies (Table 1) for 2 hours and exposed using chemiluminescence (GE Healthcare, Little Chalfont, UK). Bands were analyzed using ChemiDoc XRS (Bio-Rad) and normalized to β-actin. Data are fold change relative to room air controls ± standard error of mean (SEM).

### VEGF ELISA

Retinas were dissected as described for western blotting, tissue was sonicated, and protein concentration was determined using the Bradford method [35]. VEGF levels were measured using Quantikine ELISA for mouse VEGF (R&D Systems, Minneapolis, MN). Results are shown as pg/mL/mg total protein.

### Retinal Thickness Analysis

Eyes were enucleated and fixed in 10% neutral buffered formalin (Fisher Scientific, Pittsburg, PA) for 24 hours, then transferred to 70% ethanol or fixed in 4% paraformaldehyde for 4 hours then transferred to PBS. Eyes were paraffin embedded and retinal sections were deparaffinized with xylene and dehydrated with ethanol. Sections every 40 μm were hematoxylin and eosin stained (VWR, Radnor, PA) and mounted with Permount (Fisher Scientific). Analysis was performed using ImageJ software (NIH, Bethesda, MD). Retinal thickness measurements were described previously [36]. Briefly, two masked observers measured the entire retinal thickness, from photoreceptor outer segment tips to astrocytes, at two retinal locations, near the optic nerve (central) and in the retinal periphery. The average of two observers was reported and interclass correlation coefficient (ICC) was calculated.

### Masson’s Trichrome

Retinal cross sections were deparaffinized and post-fixed in Bouin’s fixative (Ricca Chemical, Arlington, TX) for 18 hours. Sections were rinsed in running tap water for 10 minutes. Some variations were made to the Sigma-Aldrich Masson Trichrome Kit (HT15) [37]. We chose not to use Weigert’s iron hematoxylin solution, which stains nuclei black, since the retina is highly nucleated, making it difficult to observe any collagen staining. Slides were put in Biebrich scarlet-acid fuchsin for 2 seconds and rinsed with water. Slides were differentiated in phosphotungstic/phosphomolybdic acid solution for 15 minutes, incubated with aniline blue for 10 minutes, and differentiated in 1% acetic acid for 30 seconds. After several washes, the slides were dehydrated in a series of ethanol and cleared in xylene.

### Statistical Analysis

All data were analyzed using SPSS (v.23.0; IBM Corp, Armonk, NY) or GraphPad Prism for VEGF ELISA (GraphPad Software Inc., San Diego, CA). Results are expressed as mean ± SEM. Mice from different litters (4–5 per group) were analyzed. Group differences were evaluated using ANOVA with Bonferroni’s *post hoc* comparisons. Results were considered statistically significant at p-value < 0.05.

## Results

### Hyperoxia-induced proliferative retinopathy shows severely disrupted retinal vascular development and disorganized intra-retinal angiogenesis

**Fig 1:**
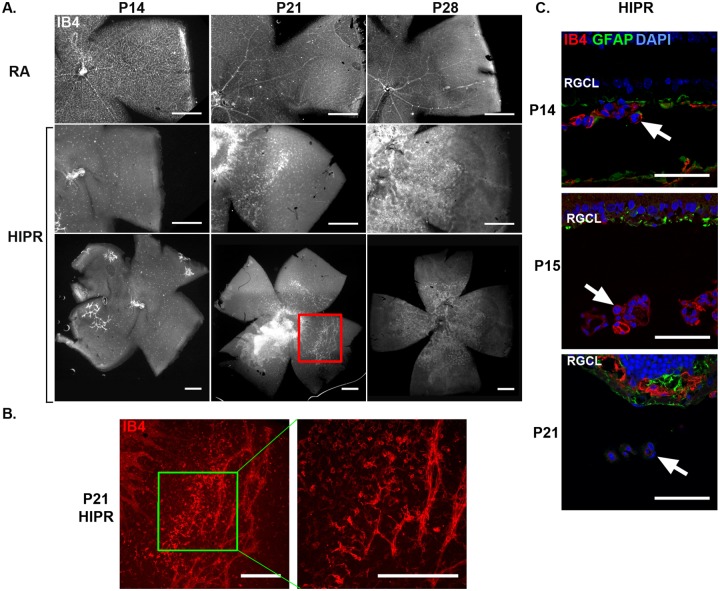
Disorganized angiogenesis in hyperoxia-induced proliferative retinopathy. (A) Representative flat mounts from P14, P21, and P28 mice in control mice (RA) and HIPR were stained with IB4 (N = 3–4). Scale bar, 500 μm. (B) Left image is an inset of the red box of P21 HIPR in (A). Right image is an inset of the green box. Persistent hyaloidal vasculature is present and attached to the retina in the mid-periphery. Scale bar, 300 μm. (C) Retinal cross sections stained with IB4 (red), GFAP (green), and DAPI (blue) of HIPR retinas show a persistent hyaloidal vessel (arrow) close to the retinal surface (N = 3–4). Scale bar, 50 μm. RGCL, retinal ganglion cell layer.

To examine retinal vascular development, we stained retinal flat mounts with isolectin B_4_ (IB4), a marker for endothelial cells, at P14, P21, and P28. Room air reared, control mice had normal retinal vascular development with the superficial network completely extending to the peripheral retina by P21 (Fig 1A). In contrast, HIPR had no evidence of normal vascular branching networks or formed blood vessels in the central or peripheral retina, replaced entirely by severely disorganized angiogenesis around the optic nerve at P21, and continuing to enlarge as a sheet of tangled endothelial cells by P28, without any signs of branching organized networks (Fig 1A). In addition, HIPR had persistent hyaloidal vessels that were attached to the retinal flat mount at the retinal mid-periphery (Fig 1B). The hyaloidal vessels may have been inadvertently removed during retinal dissections for the flat mount images (Fig 1A), so the cross sections in Fig 1C provide better examples of the persistent hyaloidal vessels. IB4 stained cross sections show persistent hyaloidal vessels in close proximity to the retina (Fig 1C). By P21, intra-retinal blood vessels form in the inner plexiform layer (IPL) (Fig 1C). Unlike what has been described as retinal revascularization in the OIR mouse model [9, 10], at P28 in the HIPR model, there was no evidence of regression of angiogenesis or revascularization of the retina nor evidence of any attempts at vascular organization or branching.

### Retinal HIF-1α, VEGF, and VEGFR2 levels increase as a result of relative hypoxia

**Fig 2:**
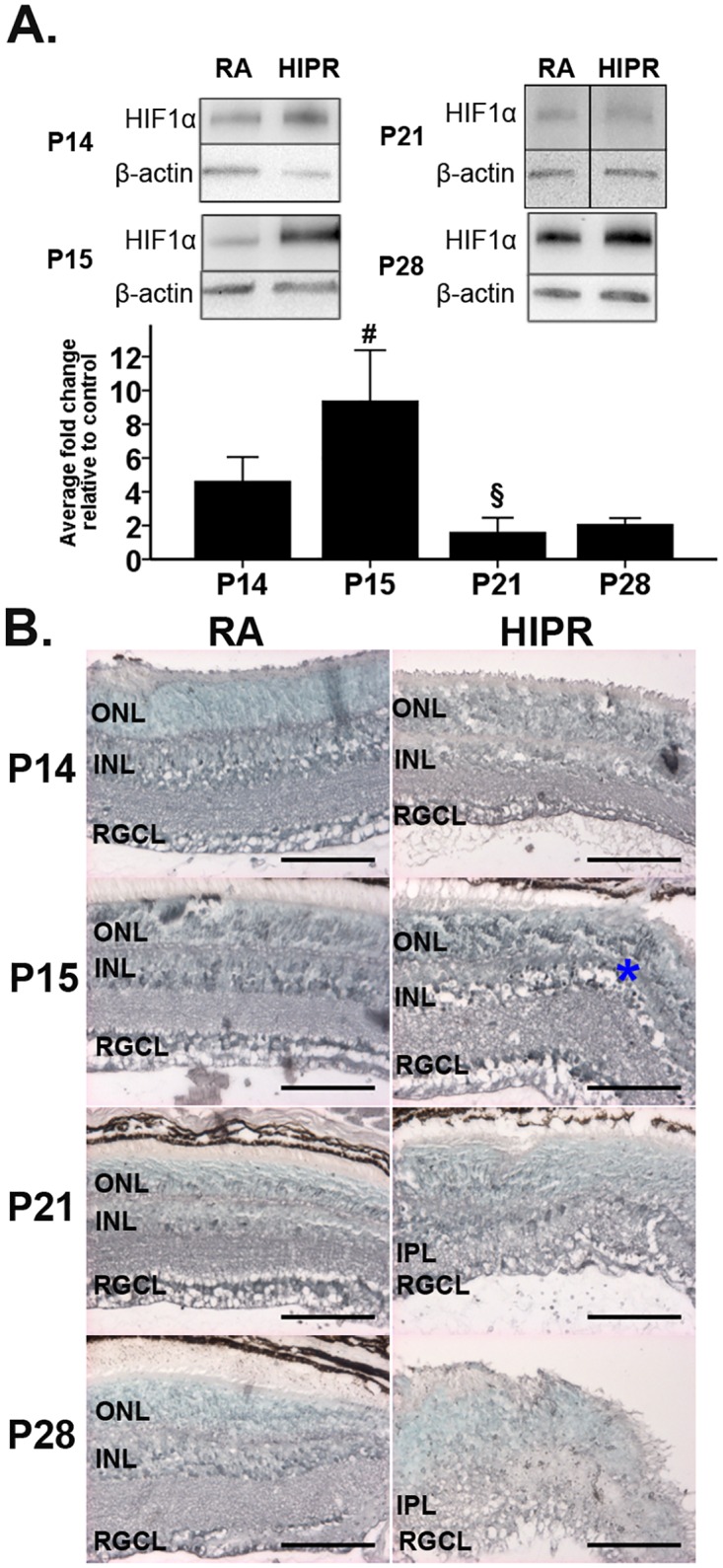
Elevated HIF-1α levels in hyperoxia-induced proliferative retinopathy. (A) Retinal HIF-1α was evaluated by western blotting, quantified by densitometry, and normalized to β-actin. Representative blots shown. HIF-1α levels significantly increased from P14 to P15 and then decreased by P21. Data were fold change ± SEM (n ≥ 4 per group). (B) Retinal cross sections were immunostained with HIF-1α (black, N = 3) and counterstained with methyl green. HIF-1α was found in the retinal ganglion cell layer and in the nuclei of the inner nuclear layer (asterisk). Scale bar, 100 μm. ONL, outer nuclear layer. INL, inner nuclear layer. RGCL, retinal ganglion cell layer. IPL, inner plexiform layer.

Given the extent of retinal vascular abnormalities in the HIPR mice, we sought to determine the HIF-1α and VEGF protein levels and their cellular locations compared to controls. Retinal HIF-1α protein expression was significantly increased in HIPR at P15 (9.4 ± 3.0 fold change), one day after removal from hyperoxia, compared to the earlier time-point at P14 (4.6 ± 1.4 fold change, p<0.05) (Fig 2A). The relative increase in HIF-1α at P15 in HIPR was no longer present by P21 (1.6 ± 0.9 fold change, p<0.05). In control retinas, HIF-1α was found in retinal ganglion cell layer (RGCL) and in the inner nuclear layer, with similar localization but higher intensity of expression seen in the HIPR mice (Fig 2B).

**Fig 3:**
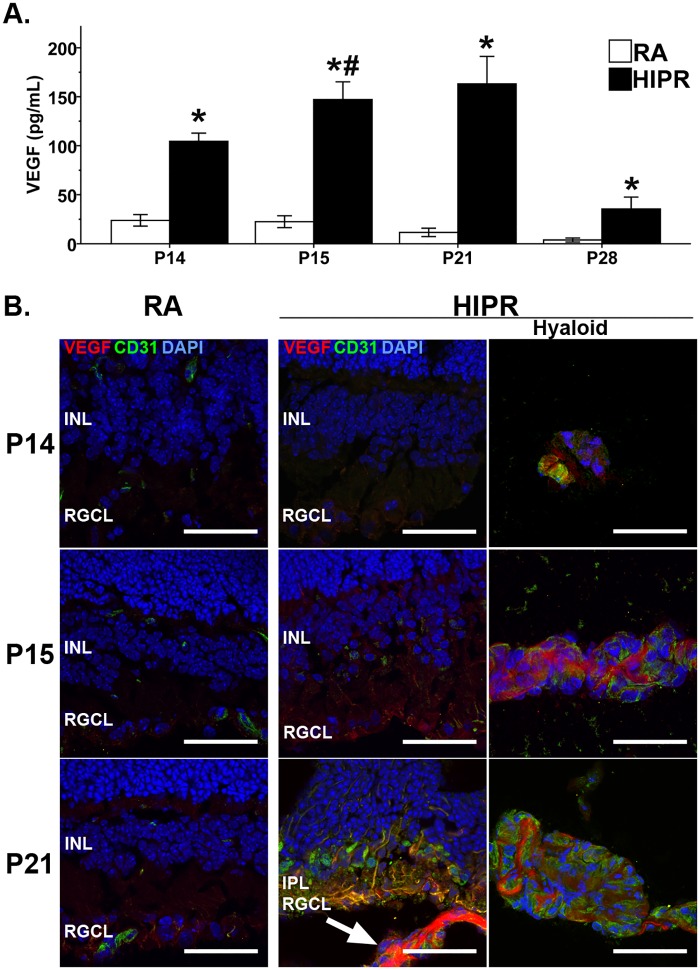
VEGF is localized to retinal ganglion cell layer and inner plexiform layer in hyperoxia- induced proliferative retinopathy retinas. (A) Retinal protein VEGF levels were quantified with an ELISA. VEGF increased until P21 and then decreased. Values were mean ± SEM (N = 4). # p<0.05 HIPR P15 compared to HIPR P14. * p<0.05 HIPR to respective age-matched RA controls. § p<0.05 HIPR P21 compared to HIPR P15. (B) Sections were immunostained with VEGF (red) and co-stained with an endothelial cell marker, CD31 (green), and DAP1 (blue, N = 2). VEGF was found in the RGCL at P15 and mainly in IPL at P21. Persistent hyaloidal vessels stained strongly for VEGF and CD31 and these hyaloidal vessels were in close proximity to the inner retina (arrow). INL, inner nuclear layer. RGCL, retinal ganglion cell layer. IPL, inner plexiform layer. Scale bar, 50 μm.

Corresponding to the increased HIF-1α protein levels, VEGF protein expression, measured by ELISA, was elevated at all time points in HIPR compared to control mice (Fig 3A). In the HIPR model, retinal VEGF levels peaked at P21 (163.1 ± 28.1 pg/ml, N = 4) and decreased by P28 (35.5 ± 12.1 pg/ml, N = 4, p<0.05) (Fig 3A). At all time points, VEGF immunostaining was present in the hyaloidal vasculature of HIPR retinas (Fig 3B). In HIPR, VEGF localized to the RGCL at P15 and by P21. VEGF in the IPL co-stained with CD31, a specific endothelial cell marker (Fig 3B). At P21, VEGF in HIPR did not co-localize with either amacrine, horizontal cells, or activated Mϋller cells (S1 Fig).

**Fig 4:**
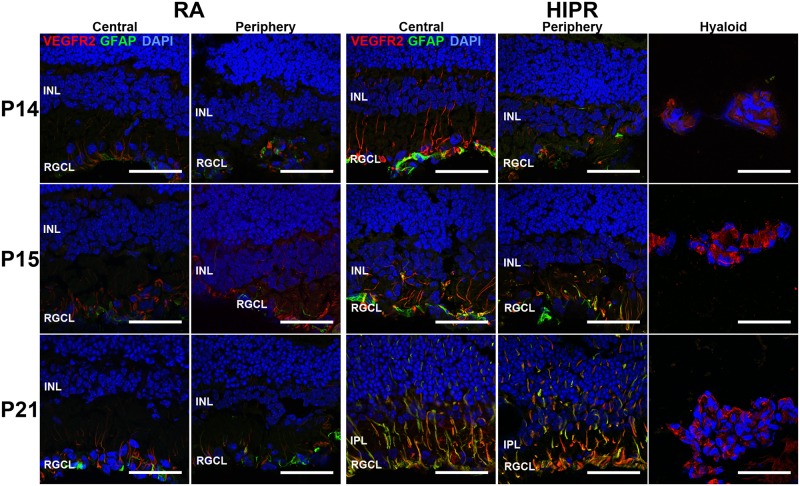
Retinal VEGFR2 levels increased in hyperoxia-induced proliferative retinopathy. Immunohistochemistry revealed VEGFR2 (red) cellular locations. Sections were stained with GFAP (green) for activated Mϋller cells (N = 3). In HIPR, VEGFR2 was co-localized with GFAP by P21. High intensity VEGFR2 staining was seen in the persistent hyaloidal vessels in HIPR. INL, inner nuclear layer. RGCL, retinal ganglion cell layer. IPL, inner plexiform layer. Scale bar, 50 μm.

Since VEGF receptor-2 (VEGFR2) is necessary for angiogenesis and a target of VEGF, we sought to determine any changes of VEGFR2 in HIPR retinas [23, 38, 39]. In adult retinas, VEGFR2 is expected to label endothelial cells, Mϋller cells, and photoreceptors [23, 25, 26], but during development VEGFR2 is found in neural cells (mainly ganglion cells) by P5 and in both retinal ganglion cells and endothelial cells by P15 [24]. Retinal cross sections were immunostained for VEGFR2, co-labeled with glial fibrillary acidic protein (GFAP), which stains for activated Mϋller cells and astrocytes. We found VEGFR2 mainly in the RGCL in the control retinas but also in some Mϋller cells (Fig 4). In HIPR, VEGFR2 staining was increased in Mϋller cells compared to controls and the staining increased over time as the model progressed (Fig 4). By P21, VEGFR2 was primarily co-stained with activated Mϋller cells (Fig 4).

### Retinal NADPH oxidase 2 expression is increased in hyperoxia-induced proliferative retinopathy

**Fig 5:**
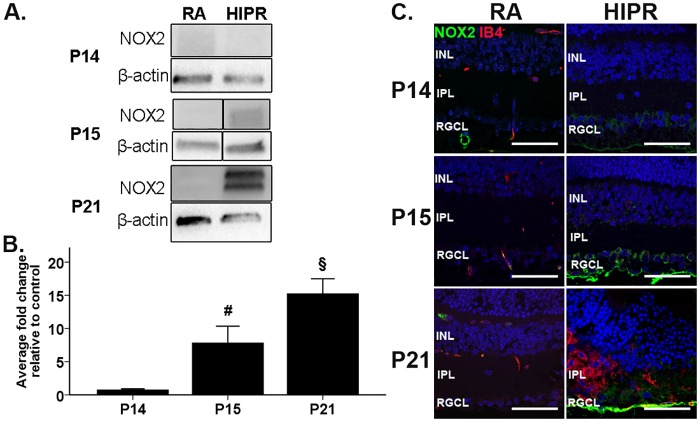
Increased NOX2 expression in hyperoxia-induced proliferative retinopathy. (A, B) Retinal NOX2 was evaluated by western blotting, quantified by densitometry, and normalized to β-actin. (A) Representative western blots are shown. The vertical black line indicates separate samples were run between these lanes on the same blot. (B) Quantification of westerns show that NOX2 levels significantly increased from P14 to P15 and from P15 to P21. Data were fold change ± SEM (n ≥ 5 per group). * p<0.05 HIPR to respective age-matched RA controls. § p<0.05 HIPR P21 compared to HIPR P15. (C) Retinal cross sections were immunostained with NOX2 (green), IB4 (red), and counterstained with DAPI (blue, N = 3). In RA, NOX2 was only seen in blood vessels. In HIPR, NOX2 was observed in the retinal ganglion cell layer. Scale bar, 50 μm. INL, inner nuclear layer. IPL, inner plexiform layer. RGCL, retinal ganglion cell layer.

In the mouse OIR model, NADPH oxidase 2 (gp91phox/NOX2) is thought to generate reactive oxygen species that promote VEGF-mediated retinal neovascularization [40, 41]. Given the extent of disorganized angiogenesis in the retinal flat mounts (Fig 1) and the increased VEGF and VEGFR2 we observed (Figs 3 and 4), we examined NOX2 protein levels and the cellular location of NOX2. In HIPR, NOX2 increased significantly from P14 (0.8 ± 0.2 fold change, N = 8) to P15 (7.8 ± 2.5 fold change, N = 5, p<0.05) and from P15 to P21 (15.3 ± 2.2 fold change N = 5, p<0.05) (Fig 5A and 5B), following the increase in VEGF (Fig 3B). NOX2 was localized to the blood vessels in control retinas. In contrast, in HIPR, NOX2 was observed in the RGCL at P15 and P21 (Fig 5C).

### Retinal thinning and persistent hyaloidal vessels in hyperoxia-induced proliferative retinopathy

**Fig 6:**
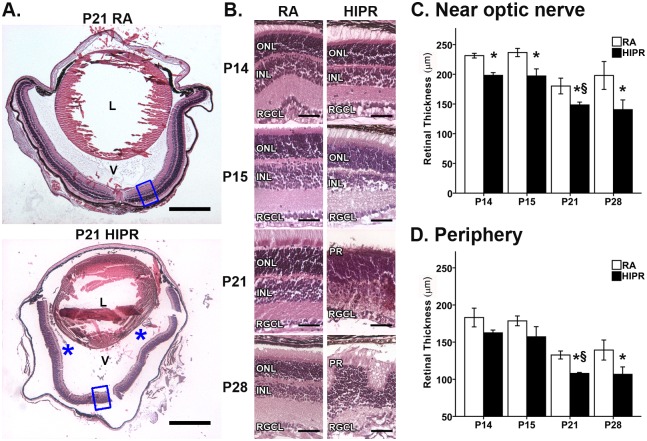
Retinal thinning near the optic nerve and in the peripheral retina in hyperoxia-induced proliferative retinopathy. (A) Cross sections of P21 RA (top) and HIPR (bottom) showing persistent hyaloidal vessels (blue asterisk). Insets (blue rectangles) correspond to higher magnification images in (B). Scale bar, 500 μm. L, lens. V, vitreous. (B) Cross sections were H&E stained (N = 3–4). In HIPR at P21 and P28, the outer nuclear layer and inner nuclear were not distinguishable, indicating severe thinning of the outer plexiform layer. Scale bar, 50 μM. ONL, outer nuclear layer. INL, inner nuclear layer. RGCL, retinal ganglion cell layer. PR, photoreceptors. The thickness of retinal layers at 100 μm from the optic nerve (C) or in the retinal periphery (D). (C) Near the optic nerve, HIPR had thinner retinas at all time points compared to RA. (D) At P15, P21, and P28, the peripheral HIPR retinas were significantly thinner than RA. * p<0.05 HIPR to respective age-matched, RA. § p<0.05 HIPR P21 compared to HIPR P15. Values were mean ± SEM (N = 3–4).

To quantify the retinal thickness and examine morphologic differences, we stained retinal cross sections with hematoxylin and eosin. Persistent hyaloidal vasculature was seen in HIPR mice at all time points; a representative P21 example is shown (Fig 6A). There was significant thinning of the outer plexiform layer by P21 and P28, with apparent merging of the inner and outer nuclear layers (Fig 6B).

To determine retinal thickness, measurements were taken from the optic nerve (central) and the periphery. At each time point, HIPR mice had significantly thinner retinas than controls in the central retina (Fig 5C, p<0.05). By P28, control central retina measured 208.5 ± 19.6 μm (N = 3, ICC = 0.95); while HIPR measured 149.5 ± 11.2 μm (N = 3, p<0.05, ICC = 0.94) (Fig 6C). In the periphery, HIPR had significantly thinner retinas than controls at P21 and P28 (Fig 6D, p<0.05). By P28, control peripheral retinas measured 146.2 ± 7.8 μm (N = 3, ICC = 0.90), compared to 106.6 ± 10.0 μm (N = 3, ICC = 0.88, p<0.05) in HIPR (Fig 6D).

### Intra- and extra-retinal fibrinogen deposition in proximity to disorganized angiogenesis

**Fig 7:**
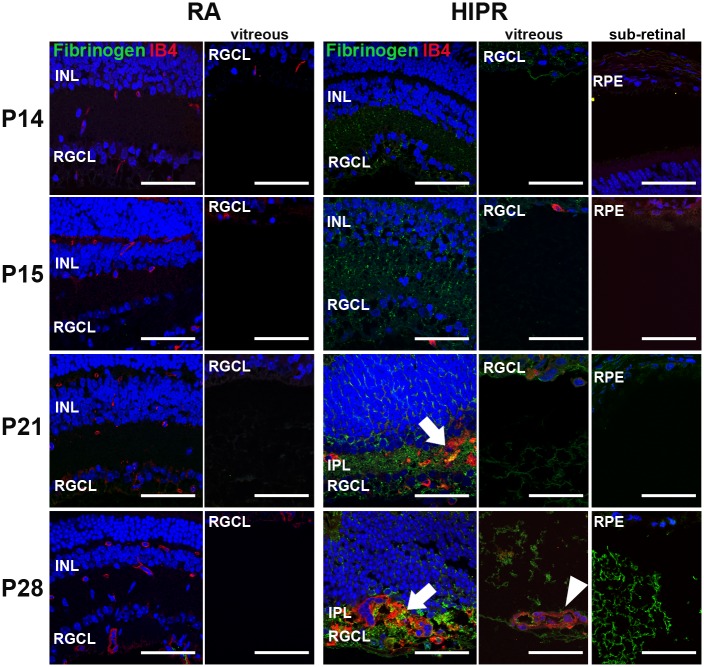
Fibrinogen present in the inner retina in hyperoxia-induced proliferative retinopathy. (A) Retinal cross sections were immunostained with fibrinogen (green), IB4 (red), and counterstained with DAPI (blue, N = 3). No fibrinogen staining was seen in the retina or the vitreous of RA mice. However, fibrinogen staining was visible in the inner nuclear layer, inner plexiform layer (arrow), and ganglion cells in HIPR. Fibrinogen staining was also observed in the vitreous of HIPR at P21 and P28, and in the sub-retinal space at P28 HIPR. Arrowhead indicates persistent hyaloidal vasculature. Scale bar, 50 μm. INL, inner nuclear layer. IPL, inner plexiform layer. RGCL, retinal ganglion cell layer. RPE, retinal pigment epithelium.

Fibrinogen immunostaining is absent from normal, healthy retinas. Fibrin and fibrinogen deposition in the lungs are seen in other BPD animal models and infants as markers of inflammation and fibrinolysis [42–46]. Retinal fibrinogen deposition is also seen in animal models with retinal ischemia, including OIR and diabetic retinopathy in monkeys [47, 48]. To examine whether the aberrant intra-retinal vessels maintained their inner blood-retinal barrier function, we studied fibrinogen depositions in the retina, vitreous, and sub-retinal space using retinal cross sections co-stained with IB4. No fibrinogen staining was observed in control retina, sub-retinal space or vitreous (Fig 7). In contrast, in HIPR, fibrinogen staining was present in the retina (inner nuclear and IPL adjacent to the severely disorganized vessels located as a confluent sheet in the IPL), vitreous, and sub-retinal space beginning at P21 (Fig 7). We were unable to identify intra-retinal blood vessels (or fibrinogen) in HIPR at P14 or P15. Fibrinogen and IB4 staining co-localized in the IPL, similar to NOX2 staining at P21 (Fig 5C). This finding suggests that the aberrant vasculature in the IPL is severely incompetent allowing large molecules, such as fibrinogen (molecular weight 340 kDa), to leak into the retina [49, 50]. The fibrinogen staining in the sub-retinal space along with retinal thinning, specifically of the outer retina, are suggestive of chronic exudative retinal detachment in HIPR.

### Hyperoxia-induced proliferative retinopathy develop a delayed retinal inflammatory response

**Fig 8:**
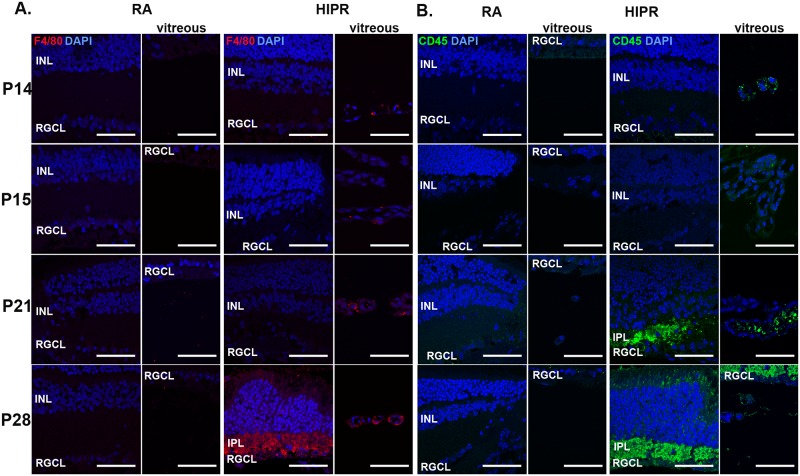
White blood cells present in the inner plexiform layer in hyperoxia-induced proliferative retinopathy. (A) Retinal cross sections were immunostained with (A) F4/80 (red) or (B) CD45 (green) and counterstained with DAPI (blue, N = 3). In RA, there was no F4/80 or CD45 staining. In HIPR, F4/80 and CD45 were both seen in the IPL at P21 and P28. Scale bar, 50 μm. INL, inner nuclear layer. IPL, inner plexiform layer. RGCL, retinal ganglion cell layer.

The presence of fibrinogen intra- and sub-retinally suggested an inflammatory response as an important component of the wound healing response and pathologic changes in this model of retinal ischemia, vascular leakage and angiogenesis [51–55]. To study leukocyte infiltration in HIPR, we performed immunofluorescence staining for F4/80 (macrophages) and CD45R (B lymphocytes and a subset of T-lymphocytes and NK cells) [30, 31]. No macrophages (Fig 8A) or CD45+ cells (Fig 8B) were seen at any of the time points in control retinas. In contrast, in HIPR, CD45R+ leukocytes were seen in the IPL at P21 and P28 (Fig 8B), closely replicating the localization of IB4, fibrinogen, and NOX2 at these time points. In addition, many macrophages were detected at P28 in the IPL (Fig 8A). Macrophages and leukocytes were observed in the persistent hyaloidal vessels of HIPR (Fig 8).

### Collagen fibers with evidence of retinal traction in hyperoxia mice

**Fig 9:**
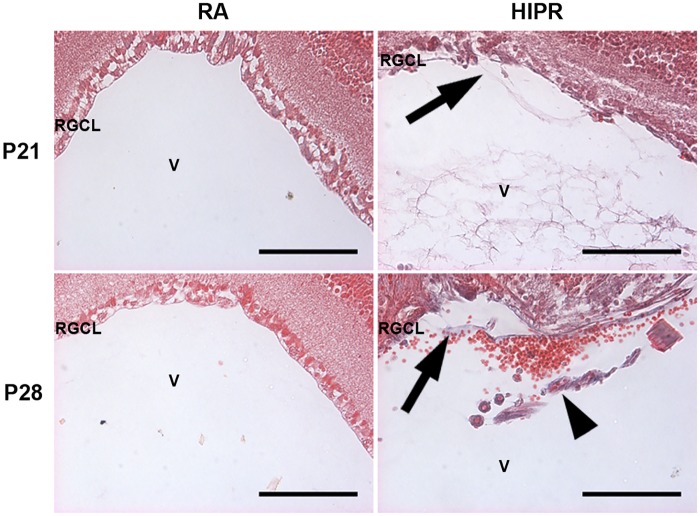
Collagen fibers found in hyperoxia-induced proliferative retinopathy. Retinal cross sections were stained with Masson’s trichrome (N = 3). Collagen fibers were detected in P21 and P28 HIPR retinas (arrows). Red blood cells were seen in the vitreous in the P28 HIPR with a nearby persistent hyaloidal vessel (arrowhead). Scale bar, 100 μm. RGCL, retinal ganglion cell layer. V, vitreous.

Since we observed evidence of retinal detachment, we stained retinal cross sections for collagen fibers using Masson’s trichrome kit to exclude tractional collagen deposition in the vitreous. The collagen fibers were only observed in HIPR (Fig 9). At P21, collagen fibers are detectable in the vitreous with distinct and focal attachment and peaking of the inner retina (arrow), suggesting that these collagen fibers are exerting traction on the retina (Fig 9). By P28, more collagen fibers are present attached to the inner retina, with evidence of vitreous hemorrhaging and persistent hyaloidal vessels (Fig 9). These results are highly consistent with traction retinal detachment.

## Discussion

**Fig 10:**
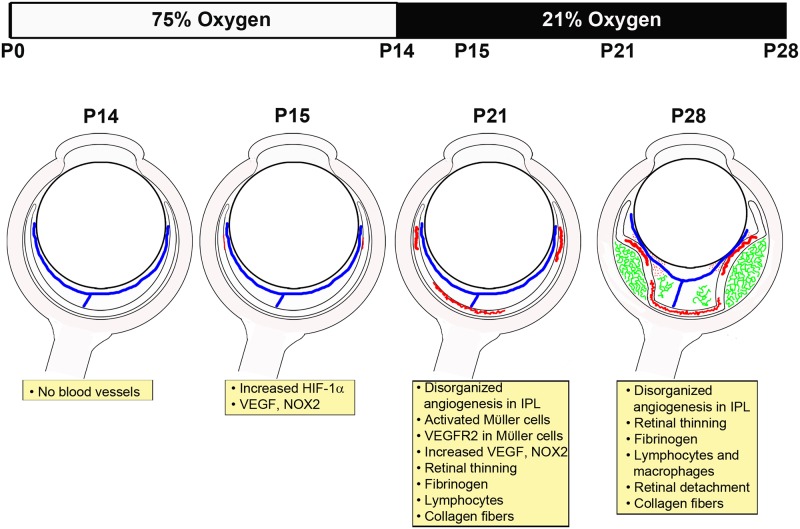
Schematic of downstream events in hyperoxia-induced proliferative retinopathy model. Upon removal from 75% oxygen at P14, there are no retinal vessels seen except for the persistent hyaloidal vessels (blue). By P15, the surge in HIF-1α leads to an increase in VEGF and angiogenesis (red) in the central retina and mid-periphery. At P21, there is disorganized angiogenesis (red) in the IPL and increased VEGF and NOX2. Fibrinogen and CD45+ lymphocytes are observed in the retina. By P28, the retina is thin and collagen fibers were detected in the vitreous, indicating traction retinal detachment. Fibrinogen (green) was found in the vitreous and sub-retinal space (suggestive of exudative detachment) and hemorrhages were seen in the vitreous (red dots). CD45+ lymphocytes and F4/80+ macrophages are increased at P28.

We have studied the retinal findings in this mouse hyperoxia-induced BPD model (HIPR), which shows severely disrupted retinal vascular development, disorganized angiogenesis, intra-retinal vascular leakage and exudative and traction retinal detachment (Fig 10). The disorganized intra-retinal blood vessels seen at P21 in the IPL do not show any semblance of normal retinal vascular patterning (Figs 1 and 7). A surge of HIF-1α, NOX2, and VEGF was observed preceding the development of this disorganized angiogenesis (Figs 2, 3, 5). The location of VEGFR2 on the activated Mϋller cells co-stained with GFAP (Fig 4) and VEGF in the IPL (Fig 3 and S1 Fig) corresponds with the location of aberrant neovascularization, suggesting that mislocalized signals from activated Mϋller cells may be driving the growth of these abnormal vasculature (Fig 4).

The mouse retinal vasculature develops entirely post-natally, preceded by an astrocyte network that forms the template for the developing superficial retinal vessels, which reaches the peripheral retina by P8 [10, 26, 56]. Around P7, the superficial blood vessels sprout downwards into the outer nuclear layer, forming the deep vascular plexus which is completed by P12, while the intermediate plexus forms by upward sprouting from the deep network between P12 and P15 in the IPL, guided by signals from the Mϋller cells [10]. The mature retinal vascular network is interconnected by P21. In HIPR, the astrocyte network appears normal at P14 and P15 and could be even seen in the peripheral retina (Fig 4B). By P21, most of the GFAP staining in the RGCL corresponds with Mϋller cells processes, suggesting that the astrocytes are no longer expressing GFAP and that they may possibly be compromised (Fig 4). In an OIR model, Dorrell et al. has shown that accelerated revascularization of the retinal vasculature and regression of intravitreal neovascularization was dependent on astrocyte and microglia survival during the vaso-obliteration phase [57]. In HIPR, severe oxygen exposure may have compromised the astrocytes, leading to loss of the blood vessels template, and may partially explain the permanent failure of development of the normally patterned superficial retinal vessels in this model.

Premature infants face a large surge in oxygen exposure at birth during their transition from the relatively hypoxic intrauterine environment to breathing room air. Subsequently they are often exposed to supraphysiologic oxygen in an effort to ensure adequate oxygen delivery to all tissues post-natally. This extra oxygen is responsible for Phase I of ROP, where the retina experiences delayed vascular development secondary to HIF down-regulation, and thus, VEGF is not released. The peripheral retina in these eyes remains avascular. Phase II begins when the retina experiences ischemia secondary to attenuated vasculature and insufficient oxygen for neural metabolism, leading to increased retinal HIF levels in the INL and RGCL [58], causing disorganized pre-retinal angiogenesis usually at the border between the avascular and vascularized zones of the retina [59]. These disorganized blood vessels, however grow into the vitreous and do not reperfuse the avascular peripheral retina, and may causes retinal traction, vitreous hemorrhage, and retinal detachment [59].

Compared to OIR, HIPR is not an exact model of ROP, due to its extreme severity. In human ROP, the retinal vasculature starts to develop *in utero* around 16 weeks gestational age [60], which is the reason we believe the HIPR phenotype has not been seen clinically in preterm infants, as 22 weeks is the earliest viability window in preterm babies [61]). In HIPR mice, oxygen exposure starts prior to retinal blood vessel development at P0, and by P14, at removal from the chamber, the retina remains completely avascular (Fig 1). At P15 (Fig 7), these retinas continue to have no evidence of retinal blood vessels, with persistent hyaloidal vasculature (Figs 1 and 6A). However, the cytokine expression profile seen in OIR is similar to what we found at P14 and P15 in HIPR. The HIF-1α and VEGF increase at P15 and P21 respectively in HIPR correlates with the second, hypoxic phase of OIR occurring at P12 in that model [10, 62] (Figs 2 and 3). The location of the neovascularization in OIR occurs in the mid-peripheral retina, between the avascular and vascularized retina, projecting into the vitreous [9, 10]. Similarly, in human ROP, angiogenesis occurs in the vitreous [59]. In HIPR, contrastingly, the neovascularization develops in the central retina within the IPL, as well as in the retinal mid-periphery at the points of contact between the hyaloidal vessels and the retina (Figs 1 and 10).

The retinal flat mounts showed peripheral avascular retina (Fig 1A) with persistent hyaloidal vessels still attached in the mid-periphery as seen on cross sections (Fig 1C). The flat mounts were stained with IB4, which has been shown to stain microglia, macrophages, and endothelial cells [63–65]. We here use it as an endothelial cell marker. We have found that while IB4 does stain macrophages in this model, the overlap with F4/80 was incomplete, indicating that IB4 was labeling endothelial cells (S2 Fig). Furthermore, using CD31, a more specific endothelial cell immunostain, we show that it similarly labels the angiogenic lesions within the IPL (Fig 3).

Given the intra-retinal location of the abnormal new vessels, we sought to determine the location of VEGFR2 in HIPR. VEGFR2 has been shown to be present in neuronal cells [25, 66, 67] and is expressed in Mϋller cells up to adulthood, indicating a role in neuronal cell protection. In HIPR, there is an increase in VEGFR2 compared to controls and a progressive increase in VEGFR2 labeling of Mϋller cells over time (Fig 4). In addition, the hyaloidal vessels stained for VEGFR2 (Fig 4) and VEGF (Fig 3), suggesting that there are actively proliferation endothelial cells in the hyaloidal vessels [68]. This could be explained by two potential mechanisms. The first potential is that the Mϋller cell (and hyaloidal) VEGFR2 expression, responding to the increase in VEGF, in an attempt to recapitulate normal vascular development and guide the blood vessels to develop the retinal vascular plexuses. However, unlike normal retinal development where neurons are sequentially maturing with a gradient of hypoxia that allows a patterned and regulated vascular development, in HIPR the retinal neurons are almost fully developed by P14, hence these signals are likely uncoordinated and lead to disorganized angiogenesis within the IPL and on the surface of the retina in contact with the hyaloid vessels in the mid-periphery. This hypothesis is supported by the VEGF immunostaining in the hyaloid (Fig 3) and Mϋller cells (S1 Fig); however additional molecular studies (e.g. *in situ* hybridization) are needed to determine the exact cellular source of VEGF in HIPR. The second potential explanation could be that the increase in VEGFR2 in the Mϋller cells is a protective mechanism for the extremely ischemic retina. Based on other studies in retinal degeneration models [69–71], it is plausible that the Mϋller cells are releasing VEGFR2 as a protective reactive signal. Similarly, VEGFR2 was found to be increased in stage 5 human ROP fibrovascular membranes [72] and soluble VEGFR2 was increased in the vitreous fluid of patients with serous retinal detachment [73]. In HIPR, it is possible that the increase in VEGFR2 could be a manifestation of both mechanisms; a guidance cue to attempt to revascularize the outer retina, as well as a protective reactive mechanism, similar to a wound healing reaction [74, 75].

To further determine the consequences of the blood vessels in the IPL, we sought to characterize whether these vessels maintained an intact blood retinal barrier. Under physiological conditions, fibrinogen is found in the plasma at low levels. However, after injury or endothelial cell dysfunction, vessel leakage, inflammatory markers, and fibrinogen levels increase in the plasma [29]. In our model, fibrinogen was found within the retina, adjacent to the aberrant blood vessels in the IPL, indicating vessel leakage (Fig 7). Fibrinogen has been shown to play a central role in inflammation [76, 77]. It is able to bind integrin and non-integrin receptors on platelets to facilitate wound healing responses including platelet aggregation, macrophage activation, chemokine expression, endothelial cell adhesion, migration, capillary formation, and mast cells adhesion and activation [29, 77]. It is likely that in our model, fibrinogen (seen at P21) contributed to macrophage activation and recruitment, first detected at P28, as well as lymphocytes, starting at P21, indicating a concurrent inflammatory response in the IPL (Fig 8).

Retinal detachment developed between P21 and P28 in this model, as shown by outer retinal thinning, photoreceptor disruption, and fibrinogen deposition in the sub-retinal space (Figs 6 and 7). There are two possible explanations for the retinal detachment in this model. The first is inflammatory, exudative detachment. Evidence supporting this include the presence of sub-retinal fibrinogen, intra-retinal exudation, and a dense inflammatory intra-retinal leukocytic infiltrate. The second potential etiology is traction. Based on the Masson’s trichrome staining of the inner retina, we believe that traction is also contributing to the retinal detachment in this model (Fig 9). Most likely, the retinal detachment is a result of a combination of both mechanisms, with inflammatory deposition as well as a progressive tractional component in the vitreous. This is not unusual and is also a commonly encountered combination of findings in infants with advanced stages of ROP [78]. The retinal thinning was characteristic of other retinal detachment models that show a dramatic loss of outer nuclear layer cells and photoreceptor cells within 1–3 days after detachment, as a result of separation from their choroidal source of oxygen [79, 80]. We also observed a loss of the outer plexiform layer (Fig 6), suggesting secondary attenuation of neuronal synapses between horizontal and bipolar cells and photoreceptor axons. In a cat model of rhegmatogenous retinal detachment, the outer plexiform layer retracted 50 days after detachment, and Mϋller cells filled the space of the degenerating photoreceptors [80]. In HIPR, Mϋller cell activation is first seen at P21 (Fig 4) along with outer plexiform layer loss (Fig 6), indicating the rapid and severe consequences of retinal detachment in this model, compared to the rhegmatogenous model. However, more experiments are necessary to confirm loss of synapses and delineate the glial response as well as the earlier time points and course of detachment in the HIPR model.

We have previously shown that mice exposed to this model have evidence of increased oxidative stress and that a mitochondrially-targeted antioxidant can prevent end organ damage in the lung and cardiovascular system [16, 81]. The NOX family is considered an important source of ROS in blood vessels and has been shown to be important in the pathogenesis of retinal angiogenesis and ischemic retinopathies [27]. Endothelial cells express NOX1, NOX2, and NOX4; while vascular smooth muscle cells express NOX1 and NOX4 [27, 28]. Mice deficient in NOX2 have reduced neovascularization in OIR due to decreased superoxide production [40]. In another study, the protein expression of NOX2 increased at P14 in OIR mice, corresponding to the onset of aberrant angiogenesis, while treatment with the pan-NOX inhibitor, apocynin, normalized VEGF expression levels and suppressed neovascularization [82]. In HIPR, increases in NOX2 were seen at P15 and P21 (Fig 5) preceding and coincident with the aberrant vascularization seen at P21, which indicated that increased NOX2 may be promoting angiogenesis in HIPR similar to its role in the OIR model.

In summary, our study showed that prolonged high oxygen exposure immediately after birth in mice to model BPD caused an aggressive form of proliferative retinopathy (HIPR), characterized by severe disruption of normal vascular development, replaced by a progressively growing sheet of disorganized intra-retinal angiogenesis from P14 to P28, along with inner retinal inflammatory reaction and ultimately a combined traction and exudative retinal detachment. The cellular components and source of VEGF in this model appear to localize to inflammatory cells and VEGFR2 expressed on Mϋller cells. The misguided attempts at retinal revascularization by Mϋller cells in this model do not recapitulate normal patterned retinal vasculature, since it occurs in the IPL in response to a local wound-healing, fibrinogen mediated reaction, rather than retinal neural elements or neuroglia. It is possible that these cells are transformed glia or endothelial cells, and further investigation using this newly developed HIPR model can help enhance our understanding of the molecular processes driving ROP and other ischemic retinopathies. Future studies focusing on intermediate time points between P15 and P21 may also help elucidate other molecular changes that promote angiogenesis and its transition to fibrosis, while long-term studies are needed to further characterize the retinal detachment of HIPR.

## Supporting Information

S1 Fig VEGF is localized to the inner plexiform layer in hyperoxia-induced proliferative retinopathy. Immunohistochemistry revealed VEGF (red) cellular locations. Sections were stained with Pax6 (yellow), a marker for amacrine, horizontal, and activated Mϋller cells and GFAP (green), a marker for activated Mϋller cells. In HIPR, VEGF was localized to the IPL but not to the GFAP+ or PAX6+ cellular elements. The far right column shows the VEGF staining separate from the VEGF and GFAP staining. INL, inner nuclear layer. RGCL, retinal ganglion cell layer. Inner plexiform layer, IPL. Scale bar, 50 μm.
(TIF)

S2 Fig Isolectin B_4_ partially co-localized with F4/80+ macrophages in hyperoxia-induced proliferative retinopathy. Immunohistochemistry of F4/80 (green), IB4 (red), and DAPI (blue) revealed some overlap of cells stained by IB4 and F4/80, indicating some IB4+ cells are macrophages at P28 in HIPR. IB4 stained additional cellular elements in the inner retina likely a combination of endothelial cells and microglia. INL, inner nuclear layer. RGCL, retinal ganglion cell layer. Inner plexiform layer, IPL. Scale bar, 50 μm.
(TIF)
