# Supplementary figures and images for: Hyperoxia-Induced Proliferative Retinopathy: Early Interruption of Retinal Vascular Development with Severe and Irreversible Neurovascular Disruption
Source: PLoS One. 2016 Nov 18;11(11):e0166886. doi: 10.1371/journal.pone.0166886 (PMC5115836; doi:10.1371/journal.pone.0166886)

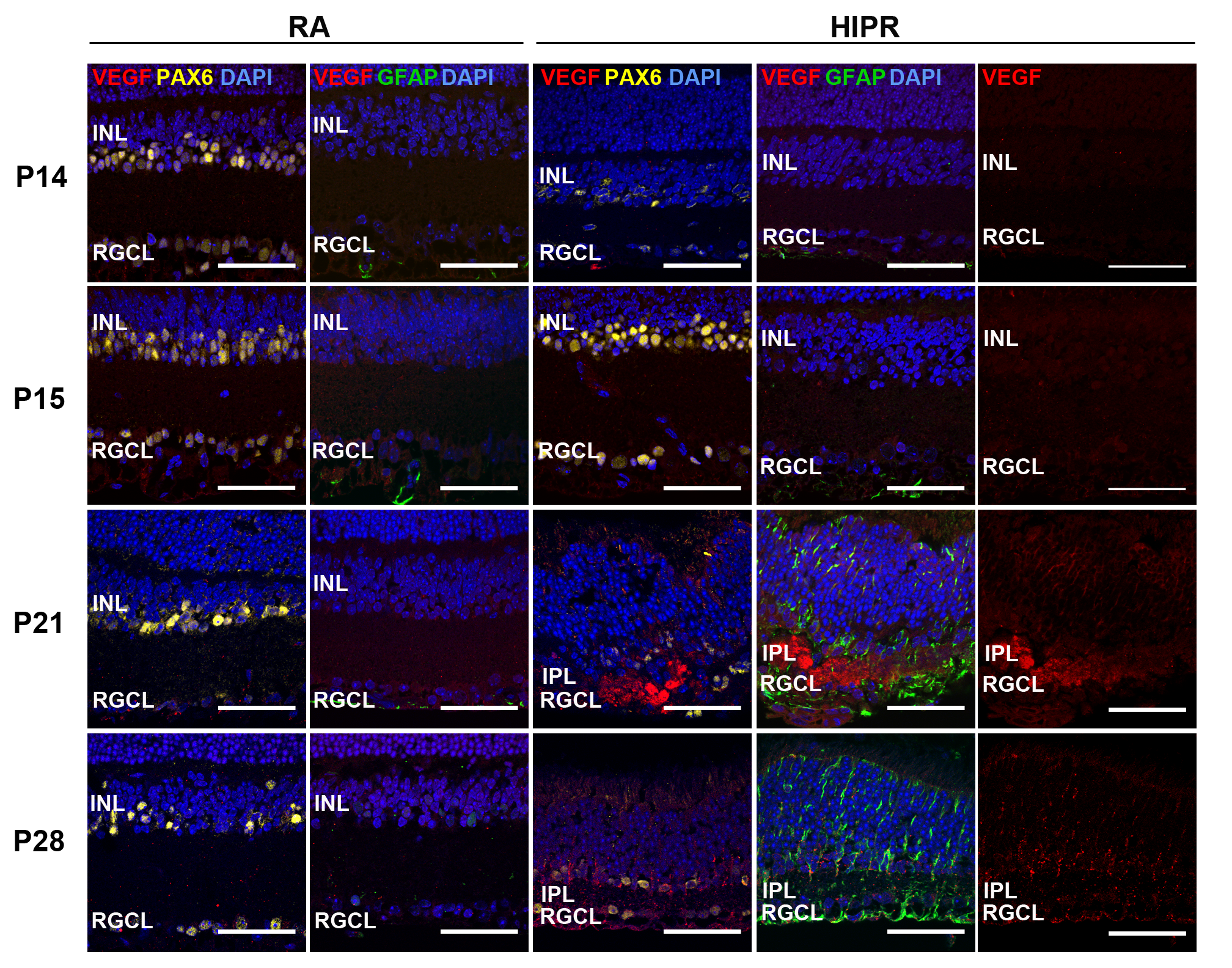

Supplement: S1 Fig — Immunohistochemistry revealed VEGF (red) cellular locations. Sections were stained with Pax6 (yellow), a marker for amacrine, horizontal, and activated Mϋller cells and GFAP (green), a marker for activated Mϋller cells. In HIPR, VEGF was localized to the IPL but not to the GFAP+ or PAX6+ cellular elements. The far right column shows the VEGF staining separate from the VEGF and GFAP staining. INL, inner nuclear layer. RGCL, retinal ganglion cell layer. Inner plexiform layer, IPL. Scale bar, 50 μm. (TIF) [file pone.0166886.s001.tif]

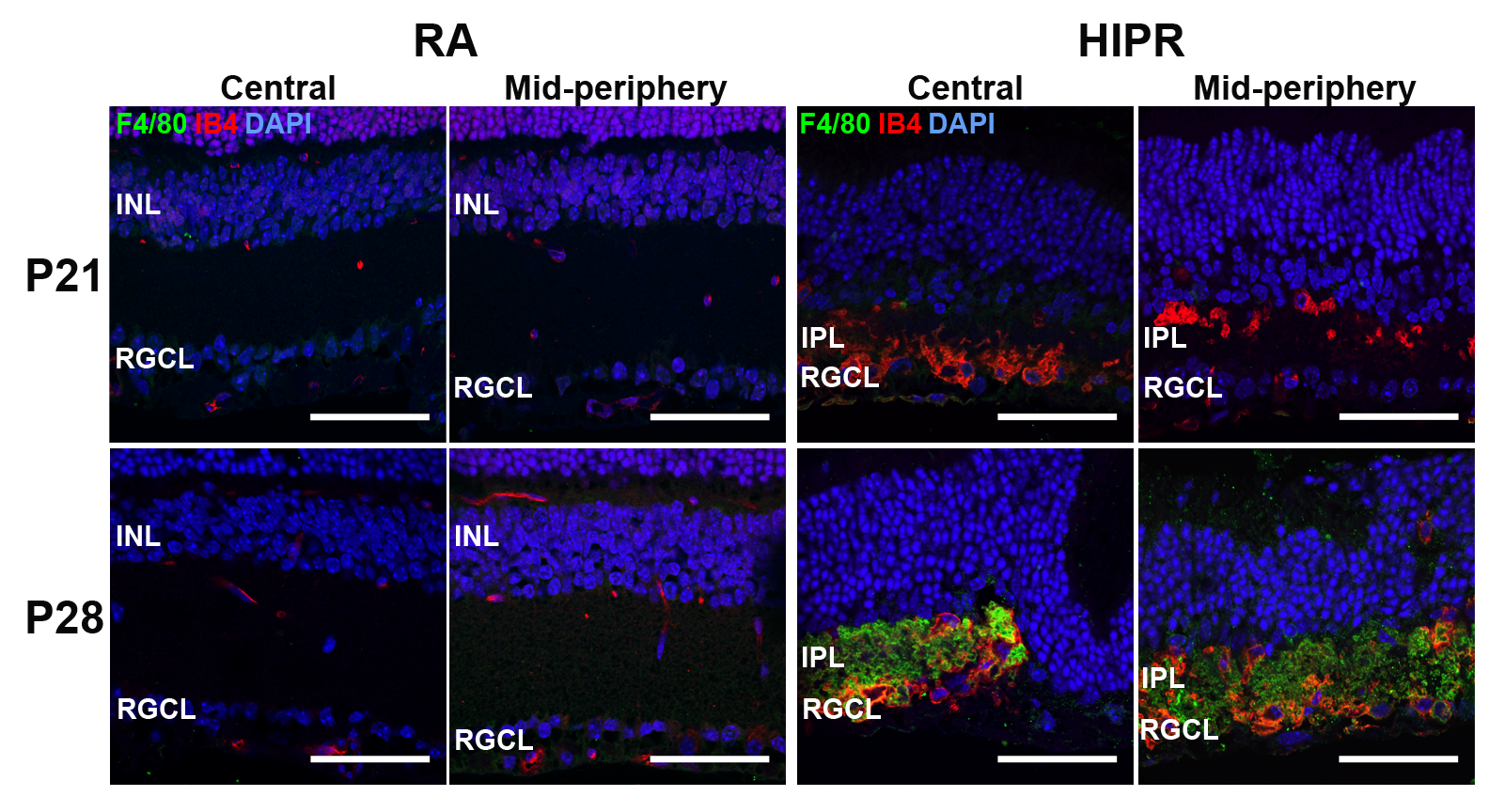

Supplement: S2 Fig — Immunohistochemistry of F4/80 (green), IB4 (red), and DAPI (blue) revealed some overlap of cells stained by IB4 and F4/80, indicating some IB4+ cells are macrophages at P28 in HIPR. IB4 stained additional cellular elements in the inner retina likely a combination of endothelial cells and microglia. INL, inner nuclear layer. RGCL, retinal ganglion cell layer. Inner plexiform layer, IPL. Scale bar, 50 μm. (TIF) [file pone.0166886.s002.tif]
